# Knowledge and Attitude on Obstetric Effects of Female Genital Mutilation among Maasai Women in Maternity Ward at Loitokitok Sub-County Hospital, Kenya

**DOI:** 10.1155/2018/8418234

**Published:** 2018-08-01

**Authors:** Keddy Wanjiru Muchene, Irene Gacheri Mageto, Joyce Jebet Cheptum

**Affiliations:** ^1^Loitokitok Sub-County Hospital, P.O. Box 28-00209, Loitokitok, Kenya; ^2^School of Nursing Sciences, University of Nairobi, P.O. Box 19676-00202, Nairobi, Kenya

## Abstract

**Background:**

Female genital mutilation (FGM) is one of the most harmful traditions still practiced in many parts of the developing world, including Kenya. The practice leads to permanent and irreversible health damages; however, knowledge and attitude of women towards its obstetric effects is scarce.

**Aim:**

The objective of this study was to determine knowledge and attitude of women towards obstetric effects of FGM among Maasai women.

**Methods:**

A hospital-based cross-sectional study was conducted at Loitokitok Sub-County Hospital among 64 Maasai women who had undergone FGM. Systematic sampling was employed to identify the respondents. Data were collected using a pretested semistructured questionnaire and analyzed through Statistical Package for the Social Sciences (SPSS) version 20.0 based on frequencies and percentages. Qualitative data were coded and categorized and thematic analysis was done.

**Results:**

Half of the women were knowledgeable on obstetric effects of FGM. Majority of them, 81% (*n*=52), sustained perineal tears during childbirth while 53% (*n*=34) had postpartum hemorrhage. Majority of the respondents, 81% (*n*=51), had negative attitude towards FGM and 87% (*n*=31) would not encourage their daughters to be circumcised. Most of them, 64% (*n*=23), disagreed that circumcision made one a respectable woman.

**Conclusion:**

Obstetric effects of FGM were fairly known and there was negative attitude towards FGM practice.

## 1. Background

Female genital mutilation/cutting (FGM/C) according to United Nations Children's Fund (UNICEF) is any procedure that involves partial or total removal of the external female genitalia or other injury to the female genital organs whether for cultural, religious, or other nontherapeutic reasons [1]. The procedure often performed by traditional practitioners is carried out on girls aged between 4 and 14 and also infants, women who are about to be married, and women who are pregnant with their first child or those who have just given birth [2]. The practice is carried out without anesthesia using scissors, razor blades, or broken glass, and it is a very painful procedure which sometimes leads to serious complications or death [3].

The prevalence of FGM/C ranges from 90 percent to as low as 5 percent in practicing countries and among different ethnic groups. Approximately 3 million girls are at risk of being mutilated/cut each year [1]. More than 200 million girls and women alive today have undergone FGM/C cut in the 30 countries in Africa, Asia, and the Middle East where high rates of the practice are reported [4]. Around one in five women have had the practice in Egypt, Djibouti, Eritrea, Somali, Niger, and Senegal [5]. Since certain minority groups and immigrant communities continue the practice in other countries as well, including in Europe and North America, the total number of girls and women worldwide who have undergone FGM/C is likely to be slightly higher [1].

FGM is practiced for a variety of reasons, sometimes at a certain age or alternatively as a rite of passage, often at puberty which is a time of vulnerability and change. Despite the fact that FGM has been illegal in Kenya for the last 12 years, it is still widely practiced. The Kenyan government estimates that 28 percent of all women between the ages of 15 and 49 in more than half the country's districts have undergone FGM with North Eastern region having the highest prevalence (97.1%) [6]. From a human rights perspective, FGM violates the right to health, security, and physical integrity of the person, the right to be free from torture and cruel inhuman or degrading treatment, and the right to life when the procedure results in death. The Kenya government banned the practice of FGM in 2002 and enforced the Children's Act which developed in 2001 in 2002 with the aim of eradicating the practice. In 2011, the FGM Act was developed to protect the rights of women and underage girls and also prosecution of those who performed FGM [7]. Despite the ban of FGM in Kenya, enforcement of the law is wanting owing to ignorance of the legislation and the consequences of FGM/C, cultural beliefs, and also reluctance by law enforcers in implementing the FGM Act [8].

FGM has both immediate and long-term complications [9]. Immediate complications include severe pain, shock, hemorrhage (bleeding), tetanus or sepsis (bacterial infection), urine retention, open sores in the genital region, and injury to nearby genital tissue. Long-term consequences can include recurrent bladder and urinary tract infections; cysts; infertility; an increased risk of childbirth complications and newborn deaths; and the need for later surgeries [10]. For example, Type III infibulation needs to be cut open later to allow for sexual intercourse and childbirth. FGM results in complications at birth for both mother and child, sometimes leading to death. The severe obstetric effects of FGM on the health of girls and women have been widely documented globally, regionally, and locally [11]. One of the complications resulting from FGM is obstructed labour which occurs due to acquired gynaetraesia [12].

At Loitokitok Sub-County Hospital maternity ward, out of an average of 128 women admitted every month, about 50% are women who are admitted with FGM-related complications. According to the health records, there is an increase in admissions to maternity ward due to the government policy of offering free maternity services and also the establishment of community strategy whereby the community units through the Community Health Workers (CHWs) encourage all pregnant women to deliver in health facilities under the care of skilled birth attendants. This increase has made the obstetric complications of the women subjected to FGM more evident owing to increased utilization of skilled birth attendance among the Maasai women. In the hospital, the number of incidences of FGM-related complications rose from 29 to 60 women in the months of April to September 2013, respectively (Table 1).

The aim of the study was to review existing knowledge and identify knowledge gaps and attitudes with regards to the obstetric effects of FGM among Maasai women so as to facilitate understanding and take appropriate action towards abolishing the unhealthy practice.

## 2. Materials and Methods

This was a descriptive cross-sectional study carried out in Loitokitok Sub-County hospital, Kajiado County, Kenya. Loitokitok is located at the southern tip of Rift Valley province in Kajiado County and is categorized among arid and semiarid districts in Kenya. It borders the Republic of Tanzania to the west, Taveta district to the southeast, Kajiado Central to the northwest, and Kibwezi to the east. The inhabitants of the region are mostly the Maasai community. The inclusion criteria were Maasai women who had undergone FGM and admitted for delivery to maternity ward and also willing to participate in the study. The sample size of the study was 64 women, which was obtained based on the average number of women who had undergone FGM admitted in the hospital's maternity in one month. This sample was calculated using Fischer's and Yamane's formulae. Systematic sampling technique was used where every 2nd woman in order of arrival to the maternity ward and met the inclusion criteria was interviewed. To get the sampling interval the target population was divided by the desired sample size. The researchers aimed to interview at least four respondents per day. Four pieces of paper were written and shuffled in a container. There was a random start for the first respondent where the nursing officer in-charge picked a number from pieces of paper written one to four.

A researcher-administered semistructured questionnaire which contained closed and open-ended questions was used to collect data. Data collection process took three weeks. Quantitative data were analyzed using Statistical Package for Social Sciences (SPSS) version 20.0 where percentages and frequencies were generated. Qualitative data generated through open-ended questions were coded and categorized into emerging patterns which were later grouped into emerging themes. Three themes emerged: knowledge of FGM, experience of FGM, and attitude towards FGM arose from the open-ended questions. A key score was developed to assess the level of knowledge of obstetric effects among the respondents. The variables indicating the obstetric effects were listed, and a respondent scored one if they mentioned an obstetric effect while they scored a zero if they did not mention any effect. A total of three correct responses was considered knowledgeable. Presentation of the findings was done using tables, bar graphs, and pie charts besides narrative descriptions. Ethical approval to carry out the study was obtained from Kenya Methodist University, Ethics Committee. Informed consent was sought from the participants after explaining about the study and the method of interview.

## 3. Results

A total of 64 respondents admitted to maternity ward were interviewed on their knowledge of obstetric effects of FGM and their attitude towards the practice.

### 3.1. Social and Demographic Characteristics

Most of the respondents, 53% (*n*=34), were aged between 14 and 20 years. Majority were married and resided in the rural areas. More than half of the respondents did not have any formal education, 56% (*n*=36), and most of them were housewives (Table 2).

### 3.2. Experience of FGM

During admission, a pelvic examination was carried out, and it was evident that all the respondents had undergone FGM; however, the type varied. Most of them, 73% (*n*=47), had type I where they had only the clitoris removed while others, 27% (*n*=17), had type II where the clitoris and the labia majora and minora were removed.

### 3.3. Knowledge of Obstetric Effects of FGM

**Figure 1:**
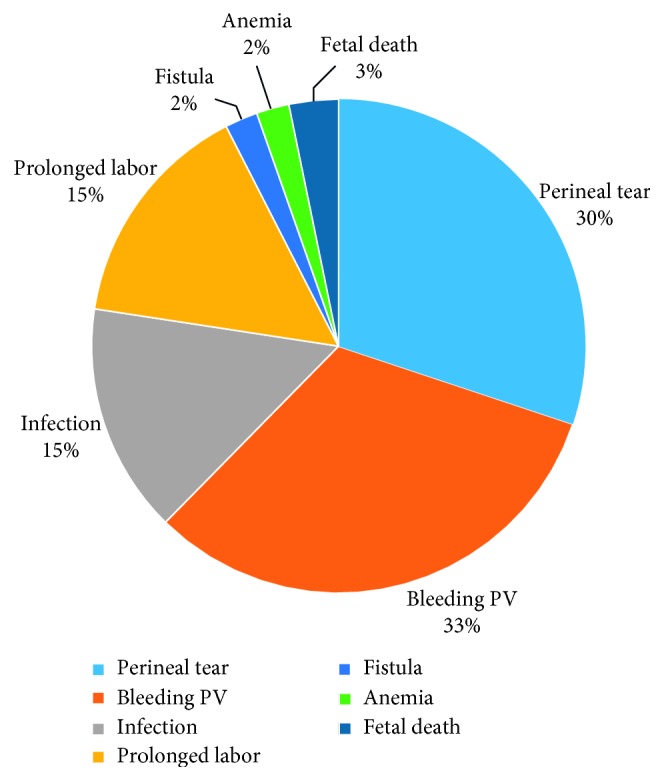
Knowledge of obstetric effects of FGM.

Irrespective of their experience on FGM, half of the respondents, 50% (*n*=32), had knowledge on the obstetric effects of FGM. Most of them mentioned per vaginal bleeding, 94% (*n*=30), followed by perineal tears, 89% (*n*=28), while prolonged labor and infection were both mentioned by 44% (*n*=14) of the respondents (Figure 1).

The 50% (*n*=32) respondents who had knowledge of obstetric complication of FGM learned about the complications during their childbirth experience after they were informed that this resulted from FGM. Most of the respondents, 78% (*n*=25), reported that if they had known the complications earlier, they could not have been circumcised, while 12% (*n*=7) said they were comfortable.

### 3.4. Attitude towards Obstetric Effects of FGM

The study's findings established that majority of the respondents, 81% (*n*=51), had negative attitude towards FGM. They thought it should be illegal and should not continue. Most, 64% (*n*=23), of the respondents disagreed with the statements that circumcision made one a woman who is respected by the community while only 33% (*n*=12) agreed with the statements. Most of the respondents, 87% (*n*=31), would not encourage their daughters to be circumcised while 14% (*n*=5) said their daughters would be circumcised. Most respondents, 67% (*n*=43), reported that FGM was not necessary and 78% (*n*=50) of them thought it was not in the woman's best interest to be circumcised.

## 4. Discussion

### 4.1. Social and Demographic Characteristics

Teenage pregnancy is itself is a risk factor of pregnancy-related complications. This situation is worsened with the presence of female genital mutilation (FGM). A factor contributing to teenage pregnancy is early marriage. The findings of the study indicated that half of the respondents were aged between 14 and 20 years and most of them were already married. These findings agree with those of the Kenya Demographic Health Survey (KDHS) which indicated a high prevalence of FGM among teenage mothers in the Maasai community [6]. This practice affects both physical maturity and change in the girls' social status. The girl is now ready for marriage and child bearing regardless of the tender age. The relationship between FGM and early marriage places the young women at a greater risk of obstetric complications such as obstructed labor.

### 4.2. Knowledge of Obstetric Effects of FGM

The study established that half of the women had knowledge on obstetric complications of FGM which is in agreement with Nasteha et al. who reported that 80.3% of Somali women had knowledge of FGM and its complications [13]. This could be attributed to access to information which leads to increase in knowledge among the communities. This could also be attributed to a change in attitude of the community towards change of behavior. People will change their behavior when they understand the hazards and indignity of harmful practices and when they realize that it is possible to give up harmful practices without giving up meaningful aspects of their culture [14, 15].

The FGM scar predisposes pregnant women to perineal tears during childbirth. The study revealed that majority of the women had perineal tears during childbirth and slightly more than half had postpartum hemorrhage. These findings are in agreement with a WHO study which established that perineal tears are a cause of obstetric hemorrhage [16]. The findings contradict the findings of a West African study on awareness of FGM complications whereby only 50% had knowledge of obstetric complications [17]. The contradiction of the findings concurs with the report which established that less than 35% of the women knew that FGM can lead to death as the result of hemorrhage [17]. These findings may indicate that the reason why many women who have undergone FGM experience obstetric complication but do not relate the complication to circumcision may be they think that it is just usual occurrence which happens to all women.

### 4.3. Attitude towards Obstetric Effects of FGM

The perception of FGM practice is dependent on the values and beliefs held by the community and also the knowledge on its effects. The study established that majority of the women felt that FGM did not make a woman feel more acceptable as a respectable woman or feel happier. Similar findings were established in a review study which found out that female circumcision is not a religious requirement and neither changed the behavior of women [18]. Most respondents in this study considered the practice backward, had outlived its usefulness, had no tangible benefits, and promoted useless pride in the initiates. This indicated that attitude towards FGM was changing, and women are positive to the change of behavior. Similar findings were established in a study assessing the values associated with FGM which established that FGM is a tradition in transition [19]. Contrary findings were however established in an Egyptian study which established that the practice was highly regarded [20] and also in Nigeria where the women did not have ability to decide whether to have it done or not done [21].

Majority of women believed the practice should be stopped and reported that they would not have their own daughters circumcised. Similar findings were reported by various studies assessing the knowledge and attitude of women on FGM [1, 22, 23]. However, in the North Eastern Province, according to a United Nations report which established that most women defended FGM, 90% supported its continuation [24] and most refugee women of Somali origin living in Ethiopia had a high intention to circumcise their daughters [25]. This contradiction indicated that communities with different cultural background have conflicting perception about FGM. There FGM practice is deep-seated in culture and religious beliefs [19].

## 5. Conclusion

Based on the study findings, the respondents were fairly knowledgeable on the obstetric effects of FGM. Additionally, most of the respondents had negative attitude towards FGM despite the practice being a rite of passage in the Maasai culture.

## 6. Recommendation

There is need for the health-care providers therefore to increase knowledge of the community on obstetric effects of FGM through health education sessions. The community elders who are the law-makers of the community need to be empowered through education to embrace nonharmful cultural practices.

## Figures and Tables

**Table 1:** Obstetric complications incidences from April to September 2013 in Loitokitok Sub-County Hospital.

Type of complication	Month					
	April 2013	May 2013	June 2013	Jul 2013	Aug 2013	Sept 2013
Perineal tears	3	4	3	5	4	6
Postpartum hemorrhage	4	3	4	5	6	7
Prolonged labor	5	7	8	10	12	13
Obstructed labor	7	8	9	10	10	11
Episiotomy	5	7	9	10	13	15
Anemia secondary to postpartum hemorrhage	5	6	5	7	7	8

**Table 2:** Sociodemographic characteristics.

Characteristic	Frequency (*n*)	Percentage (%)
*Age*		
14–20	34	53.1
21–30	18	28.1
31–40	10	15.6
41–49	2	3.2

*Residence*		
Rural	50	78
Urban	14	22

*Education level*		
None	36	56
Primary	12	19
Secondary	14	22
Postsecondary	2	3

*Marital status*		
Married	51	80
Single	11	17
Separated	2	3

*Occupation*		
Formal employment	9	14
Self–employment	4	6
Farmer	14	22
Casual laborer	9	14
Housewife	23	36
Student	5	8
